# Colonic dysmotility regulated by downregulation of PDGFRα^+^ cells / SK3 channel in DSS-induced colitis mice

**DOI:** 10.1371/journal.pone.0312413

**Published:** 2024-12-17

**Authors:** Chen Lu, Hongxia Zhu, Haiqian Lu, Xianjing Xie, Ling Tong, Yujia Li, Zhida Qian

**Affiliations:** 1 The International Peace Maternity and Child Health Hospital, School of Medicine, Shanghai Jiao Tong University, Shanghai, China; 2 Shanghai Key Laboratory of Embryo Original Diseases, Shanghai, China; 3 Department of Anatomy and Physiology, Shanghai Jiao Tong University School of Medicine, Shanghai, China; Kazi Nazrul University, INDIA

## Abstract

Colitis is a complex multifactorial disease with an unknown aetiology that mainly manifests as chronic refractory colon transmission disorders. Smooth muscle, the main source of colon transmission power, consists of not only smooth muscle cells (SMCs) but also PDGFRα^+^ cells that mediate smooth muscle relaxation and ICCs that mediate contraction. PDGFRα^+^ cells and their unique small conductance Ca^2+^-activated K (SK3) channels are crucial in regulating colonic transit by exerting inhibitory effects. In this study, the contributions of the SK3 signalling pathway in PDGFRα^+^ cells to colitis-induced colonic transit dysmotility were investigated in DSS-induced colitis mice. An experiment was conducted to record the transmission of waves during smooth muscle contraction in the colon, using a colonic migrating motor complex(CMMC). Western blotting was utilized for protein expression detection, while PCR was employed for gene expression analysis. Immunofluorescence staining was used to detect the co-localization of SK3 channels with PDGFRα^+^ cells. In the colitis group, the weight of mice was reduced, the length of colon was shortened, and the disease activity index (DAI) was significantly increased. In the CMMC experiment, colon transmission was significantly disrupted in the colitis group compared to the control group, with a consistent colonic transmission amplitude and frequency. The sensitivity of mice with colitis to SK3 antagonists and agonists (apamin and CyPPA) was lower than that of the control group in CMMC experiment. The expression levels of mRNA and protein of PDGFRα and SK3 channels in colon of mice with colitis were decreased. Less SK3 channel colocalization with PDGFRα^+^ cells was observed in the colitis mouse group than in the control group. The findings indicated that colonic transit disorder in DSS-induced colitis mice is caused by the down-regulation of PDGFRα^+^ cells / SK3 channel expression.

## Introduction

In the digestive system, colonic motility plays critical roles in absorbing water, storing chyme, and propelling stool into the rectum [1]. Smooth muscle from colon tissue has important contributions to generating colon transit motility. Colitis is a recurrent and remitting disease with an increasing incidence and prevalence, accompanied by functional alterations and abnormal intestinal motility [2]. In recent years, the incidence of colitis has increased annually. Based on statistics, colitis has an incidence rate of around 12.6 per 100,000 individuals, with a recurrence rate as high as 50% [3]. Colon transit disturbances are caused by changes in neuromuscular function, involving components like the enteric nervous system (ENS) and the SIP syncytium. For example, platelet-derived growth factor receptor α positive (PDGFRα^+^) cell, interstitial cells of Cajal (ICC) and smooth muscle cell (SMC) are electrically coupled in smooth muscle tissue to coordinate the neuromodulation movement pattern in the colon [4–6]. In the study of gastrointestinal motility disorder, the important role of ICCs in colon motility has been revealed [5]. The change of ICC number was reported by Nunzia Bernardini and others, which provided a morphological basis for better understanding the movement abnormality of colitis patients [7]. Up to now, although considerable progress has been made in studying the mechanism of colonic motility disorder, little is known about the effect of PDGFRα^+^, another interstitial cell, on colonic motility disorder in patients with colitis.

PDGFRα^+^ cells, also known as "fibroblast-like cells" (FLCs), are located between the annular muscle and the longitudinal muscle next to the motor neurons in the gastrointestinal muscle [8]. PDGF receptor α (PDGFRα) and PDGF receptor β (PDGFRβ) are two important tyrosine kinase receptors among PDGF family proteins [9]. High expression of PDGFRα and SK3 channels has been detected in PDGFRα^+^ cells [10]. SK3 channels in PDGFRα^+^ cells can induce transient outwards K^+^ currents when activated by dynamic cytoplasmic Ca^2+^ signalling [10–12]. Gallegoet et al. reported that apamin can effectively inhibit SK3 channels and inhibitory junction potentials (IJPs) regulated by purinergic neurotransmitters on PDGFRα^+^ cells [8,13]. Therefore, PDGFRα^+^ cells, as postjunction cells expressing purinergic receptor P2Y1, regulate inhibitory purinergic neurotransmitters (such as ADP and ATP) released by ENS to regulate colon transmission. In addition, fast IJPs are induced by the specific conduction of purine signals in PDGFRα^+^ cells through the P2Y1 receptor [11,14]. In mice with colitis, Kumagai et al. reported that PDGFRα^+^ cells are not only important inducers of fibrosis in the recovery phase but also potential chemoattractants for mononuclear inflammatory cells involved in the active inflammatory phase [15]. However, the role of PDGFRα^+^ cells in colonic dysmotility in colitis disease has not been previously studied. Therefore, this study revealed the regulation of the SK3 signalling pathway in PDGFRα^+^ cells in mice with colitis.

## Methods and materials

### Ethical consideration

The study was conducted in strict accordance with the relevant regulations of the Guidelines for the Care and Use of Laboratory Animals (STCC Publication No. 2, Revised 1988) published by the Science and Technology Commission, P.R.C. The protocol was approved by the Committee on the Ethics of Animal Experiments of Shanghai Jiaotong University School of Medicine (Permit Number: Hu 686–2009).

### Animals

According to previous literature reports, the most widely used mouse model of colitis employs dextran sodium sulfate (DSS), a chemical colitogen with anticoagulant properties, to induce disease. DSS is a watersoluble, negatively charged, sulfated polysaccharide with a highly variable molecular weight, ranging from 5 to 1400 kDa. The DSS colitis model is very popular in IBD research due to its rapidity, simplicity, reproducibility, and controllability. Acute, chronic, and relapsing models of intestinal inflammation can be achieved by modifying the concentration of DSS and the frequency of administration. The following animal model was used: male ICR mice, 5 weeks old, clean grade, and provided by Shanghai Slack Laboratory Animal Company. Drinking water: Mice in the control group were provided ordinary pure water, while mice in the experimental group were provided pure water containing 3% DSS; namely, 3 g of DSS was added to 100 mL water, and the animals in both groups had free access to water and food. Grouping: Twenty mice were randomly divided into two groups (control group and experimental group), with 10 mice in each group, with a body weight of approximately 25 ± 3 g. The mice were fed continuously for 7 days for a cycle, the feeding temperature was controlled at 24°C, and the light cycle was alternated between day and night for 12 h. Daily observation and records: The clinical manifestations of the colitis mouse model, including weight loss, faecal laxity and blood in the stool, were noted daily, and the body weight and disease activity index (DAI) of the mice were recorded daily.

### Haematoxylin–eosin staining

Materials: Mice were anaesthetized and euthanized. The abdominal cavity was opened along the midline, and colon tissues were removed and placed in PBS solution. The muscle was cut along the longitudinal axis of the colon, and then the faeces attached to the colon were gently removed. The colon was then fully unfolded and fixed on a silicone plate with a fine needle. Fixation: Colonic tissue was fixed with 10% formalin for 24 h at 24°C. Dehydration: The formalin solution attached to the intestinal wall was removed by washing with running water, after which the tissue was dehydrated with alcohol. Finally, the tissue was placed in the transparent agent xylene, and the alcohol in the tissue was replaced with xylene. Wax immersion and embedding: The tissue was embedded in paraffin. After slicing, spreading and baking, the tissue was cut to a thickness of approximately 5 μm, spread in hot water, transferred to a slide, and dried. Finally, haematoxylin and eosin (H&E) were used for staining. A histological assessment of colitis was performed using a standardized, semiquantitative scoring system used by pathologists, i.e., pathologists who were blinded to the time point, strain, and treatment group. Briefly, three pathological tissues were scored: (1) active inflammation (granulocyte infiltration); (2) chronic inflammation (lymphocytes, plasma cells, and macrophages in the mucosa and submucosa); and (3) villus distortion (flattening and/or widening of the normal villous architecture) [16,17].

### CMMC experiments

Mice were administered general anaesthesia by inhalation of isoflurane and euthanized by cervical dislocation. Then, the abdominal cavity was opened along the midabdominal line, and the colon tissue was quickly removed and placed in a Krebs solution with continuous oxygenation. The colon, along with the mesentery, was then attached to the silicone plate with a small pin. A 1 ml syringe was filled with Krebs solution and passed through an opening at one end of the colon, after which water pressure was applied to flush out the faeces from the colon. This procedure was repeated until all faeces were removed from the colon. A ball about the size of a faecal particle is then inserted into the proximal end of the colon. The colon was placed in a thermostatic tank filled with approximately 20 mL of Krebs solution while oxygen was pumped continuously. At this time, the colon specimens were placed in Krebs solution containing the following components (all concentrations in mmol/L: NaCl 121.9; NaHCO_3_ 15.5; KCl 5.9; MgSO_4_ 1.2; KH_2_PO_4_ 1.2; glucose 11.5; and CaCl_2_ 2.4) at constant temperature for approximately 30 min to restore the contractile activity of the colon cells. Using a tension transducer, two 5/0 threads (USPs) were fixed to the proximal and distal parts of the colon with biogel, and 0.1 g of tension was applied to maintain the contractile activity of the colon. The isometric force transducer (RM6240C, Chengdu Instrument Factory, China), which records smooth muscle contractions, was then connected to a biological amplifier so that the colonic contraction waves recorded on the transducer could be displayed on a computer through the biological amplifier. Thus, the contractile characteristics of the proximal and distal colon were visually compared.

### Western blot analysis

Colon smooth muscle tissue was removed from the mouse models, weighed, and then crushed using liquid nitrogen and a mortar. RIPA buffer (3 ml of RIPA per gram of tissue; P0013, Beyotime Chemical Co., Jiangsu, China) and PMSF (30 μl per gram of tissue, 10 mg/ml PMSF; Beyotime Chemical Co., Jiangsu, China) were added to the tissue. The suspension was further homogenized (15,000 rpm for 1 min) at 4°C and incubated on ice for 30 minutes. The protein samples were transferred to a 4°C centrifuge tube and centrifuged for 15 min (15,000 rpm/min). Protein samples (40 μg/lane) were separated by electrophoresis on 10% gels (SDS‒PAGE) and then transferred from the polyacrylamide gels to PVDF membranes with a semidry transblot unit (Bio-Rad). The PVDF membrane was then blocked with 5% buttermilk and 0.1% Tris-buffered saline/Tween 20 (TBST). The primary antibody was added to the PVDF membrane and incubated at 4°C for 12 h. Then, the secondary antibody was added the next day, and the membrane was incubated at room temperature for 120 minutes. The following antibodies were used in this study: anti-PDGFRα (Cell Signaling Technology, Danvers, USA; dilution 1:1000); anti-SK3 (dilution 1:500; Abcam, Cambridge, USA); anti-tubulin (dilution 1:1000; Beyotime Chemical Co.); anti-rabbit IgG, HRP-linked (dilution 1:1000; CST Company); and anti-mouse IgG, HRP-linked (CST Company; dilution 1:1000).

### RNA extraction and reverse-transcription quantitative PCR

We first extracted RNA from smooth muscle tissue samples using an RNeasy Total RNA Kit (TIANGEN, Beijing, China). First-strand cDNA was then synthesized with a PrimeScript RT Reagent Kit with gDNA Eraser (Takara, Dalian, China). Quantitative PCR was subsequently performed using FastStart Universal SYBR Green MasterMix (Roche, Roche) on a 7500 Real Time PCR system (Applied Biosystems Waltham, MA, USA) with specific primers. Finally, the expression of target genes relative to the endogenous control was examined using the ΔCT method. The sequences of primers used were as follows: Pdgfra F-ATGACAGCAGGCAGGGCTTCAACG,

R-CGGCACAGGTCACCACGATCGTTT;

Kcnn3F-CTGCTGGTGTTCAGCATCTCTCTG, R-GTCCCCATAGCCAATGGAAAGGAAC; and

Gapdh F-GCCGATGCCCCCATGTTTGTGA,

R-GGGTGGCAGTGATGGCATGGAC.

### Immunofluorescence staining

Colonic tissues were obtained as described above. Tissue samples were fixed with 4% ice-cold paraformaldehyde for 8 h, dehydrated in 20% sucrose, embedded in frozen tissue blocks and cut into 8- to 10-μm-thick frozen sections at −24°C. The samples were incubated in 0.1 M phosphate-buffered saline (PBS) containing 10% normal goat serum for 2 h at 24°C to block nonspecific binding and incubated with a goat anti-PDGFRα antibody (1:200; AF1062, R&D Systems, USA) and a rabbit anti-SK3 (Ki67) antibody (1:50; GB13030, Wuhan Goodbio Technology, China) mixed with Triton-X100 (0.5%, Sigma–Aldrich, St. Louis, MO, USA) at 4°C for 24 h. The samples were washed with 0.1 M PBS for 30 min and then incubated at 24°C with Cy3-conjugated anti-goat IgG (1:300; GB21404, Wuhan Goodbio Technology, China), Alexa Fluor 488-conjugated goat anti-rabbit IgG (1:100, Jackson ImmunoResearch, USA) and DAPI for 2 h. Images were acquired using a confocal laser-scanning microscope (Leica TCS SP8, Germany).

### Statistical analysis

This trial was performed as a case‒control study. All of the data analyses were conducted with SPSS 17.0 software. Our data are presented as the means ± SEs. One-way analysis of variance (ANOVA) was used to analyse the differences among the groups, followed by the Bonferroni post hoc correction and Student’s unpaired t test if necessary. A p value less than 0.05 was considered to indicate a significant difference between groups, and an n value corresponded to the number of animals used in the indicated experiment.

## Results

### A mouse model of DSS-induced colitis

**Fig 1:**
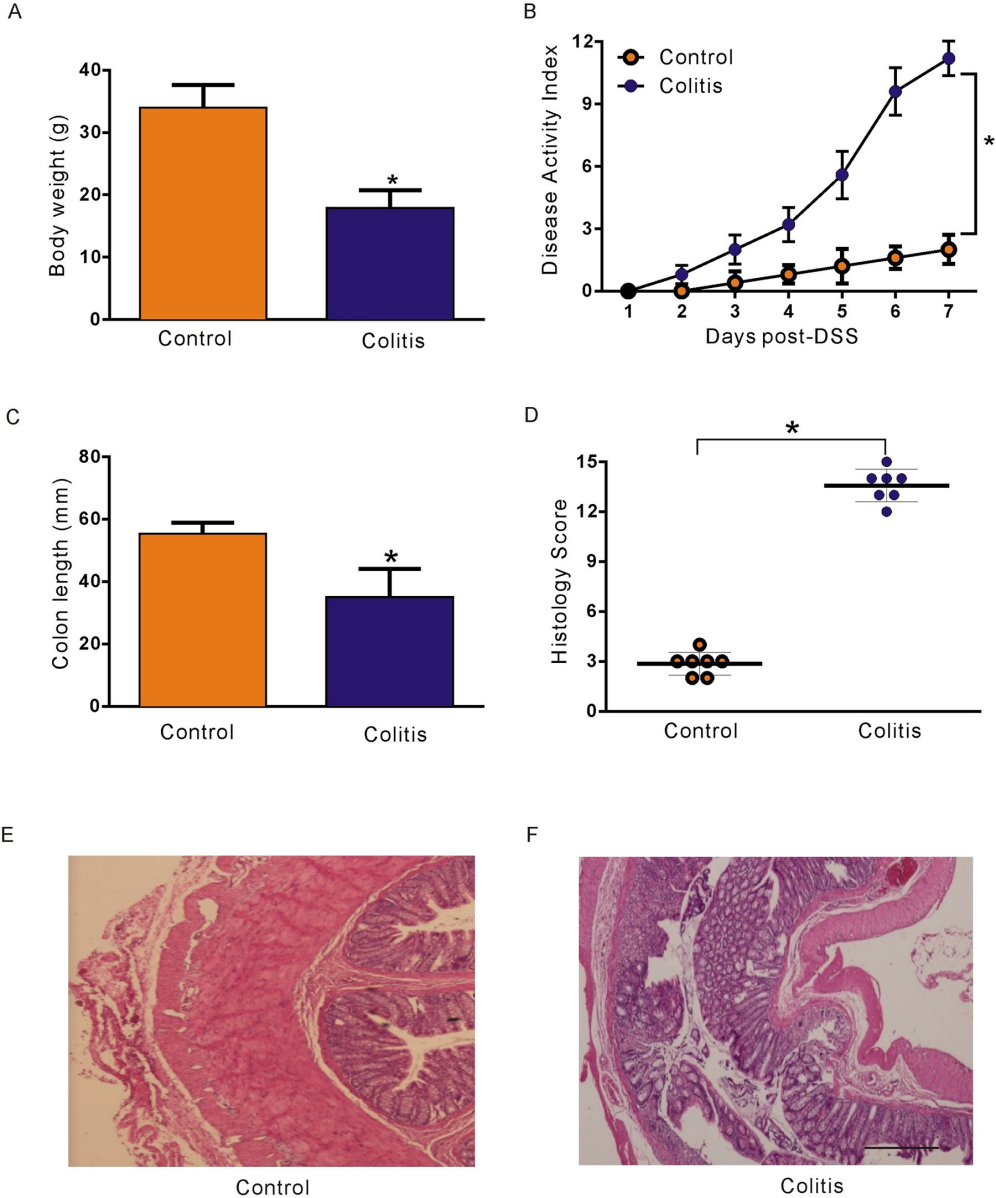
Changes in control and colitis mice. **A.** Comparison of body weights between control and colitis mice. **B.** The clinical DAI is a composite of weight change (percentage of Day 0), stool score, and occult blood index. **C.** Comparison of colon lengths between control and colitis mice. **D.** Histopathology scores of the control and colitis groups. **E-F.** Representative micrographs of H&E-stained colon tissues from control and colitis model mice 7 days after DSS treatment. Bars, 100 μm.

The experimental mice were provided 3% DSS in water for seven days. The body weight of DSS-induced colitis mice was significantly reduced (34.1 ± 1.13 g for control mice; 18.7 ± 0.81 g for colitis mice; *P* < 0.05; n = 20; Fig 1A). Then, we examined the disease activity index (DAI) of control and colitis model mice, and the results showed that the DAI of colitis model mice was significantly increased (P < 0.05; n = 7; Fig 1B). In addition, colitis mice had significantly shorter colon lengths than control mice (*P* < 0.05; n = 8; Fig 1C). The histological score (including inflammatory cell infiltration and overall tissue damage) (P < 0.05; n = 7; Fig 1D) was markedly greater in the colitis mice than in the control mice. In addition, observations of H&E staining under a light microscope revealed that DSS-induced colitis in mice was characterized by the destruction of the intestinal epithelium, abnormal crypts, oedema, and infiltration of inflammatory cells (P < 0.05; n = 7; Fig 1E and 1F), as well as haematochezia (not shown).

### Transit patterns of the CMMC in control and colitis model mice

**Fig 2:**
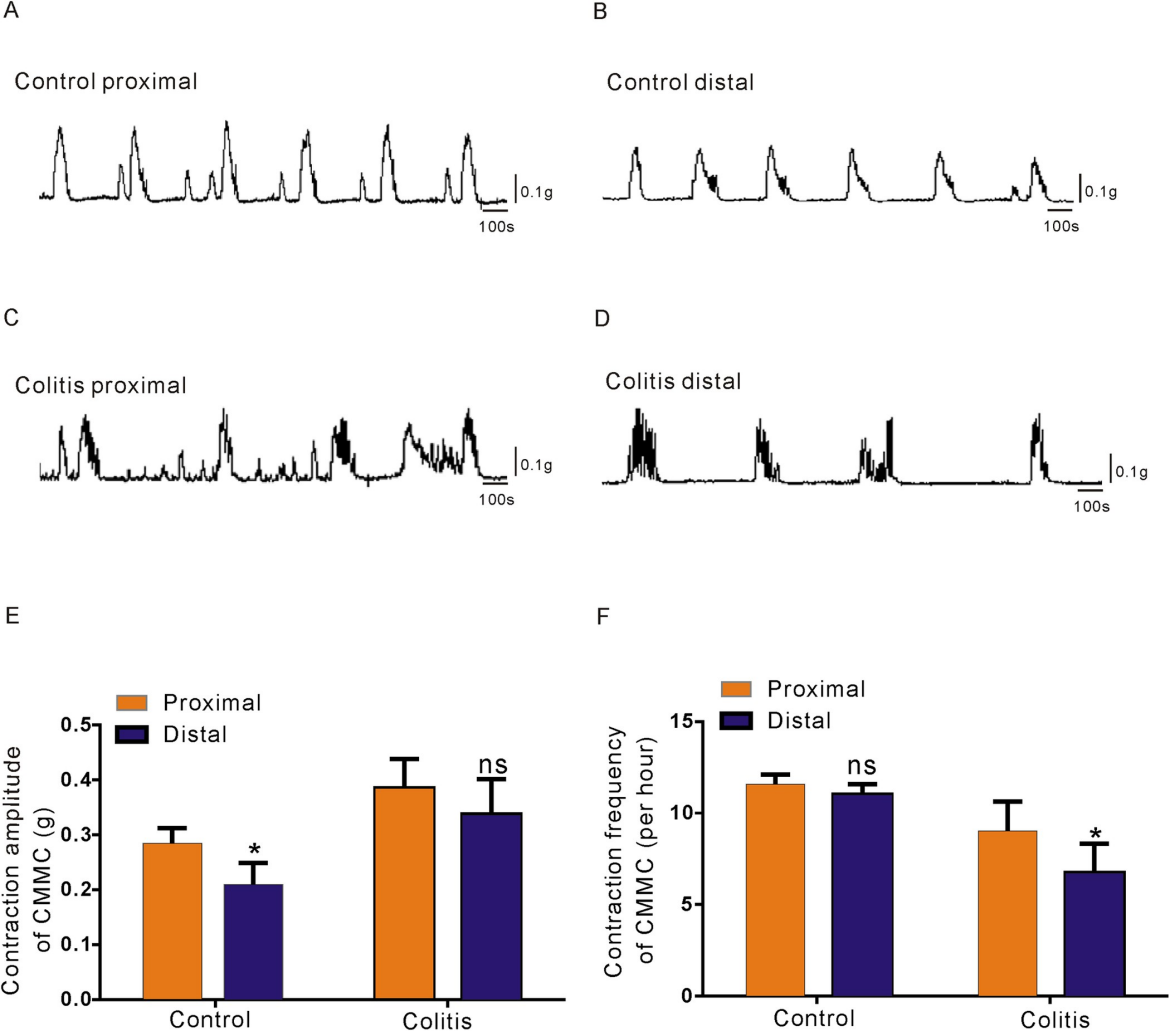
Transmission patterns of CMMCs from control and colitis model mice. **A-D.** Transmission waves of the CMMC in the proximal and distal colon of the control mice (A, B) and colitis mice (C, D). **E–F.** Comparison of the CMMC contraction tension (E) and frequency (F) between control and colitis mice.

SK3 channels on PDGFRα^+^ cells are responsible for neuro-mediated diastolic responses in gastrointestinal motility transmission [18]. As a method to further confirm the low expression of PDGFRα and SK3 channels in colitis mice, we used CMMC as the main form of dynamic colonic transmission to observe the difference between the control group and colitis group [19]. We found that the frequency and amplitude of contraction of the proximal and distal colon of the mice with colitis were irregular (*P* < 0.05; n = 8; Fig 2C and 2D), while those of the control mice were significantly consistent (*P* < 0.05; n = 8; Fig 2A and 2B). Moreover, the dynamic transmission of the colon in colitis mice was marked by irregular contraction. The transit amplitude and frequency of the CMMC in the proximal and distal colon were consistent in the control group, while the contraction amplitude and frequency of the CMMC in the proximal and distal colonic segments were significantly different in the colitis group (*P* < 0.05; n = 8; Fig 2E and 2F). Compared with the strong to weak transit amplitude from the proximal to distal colon and the consistent frequency in the control group, the transit frequency and amplitude of the CMMC in the colitis group were obviously irregular.

### Effect of the SK3 channel antagonist in PDGFRα+ cells on CMMCs

**Fig 3:**
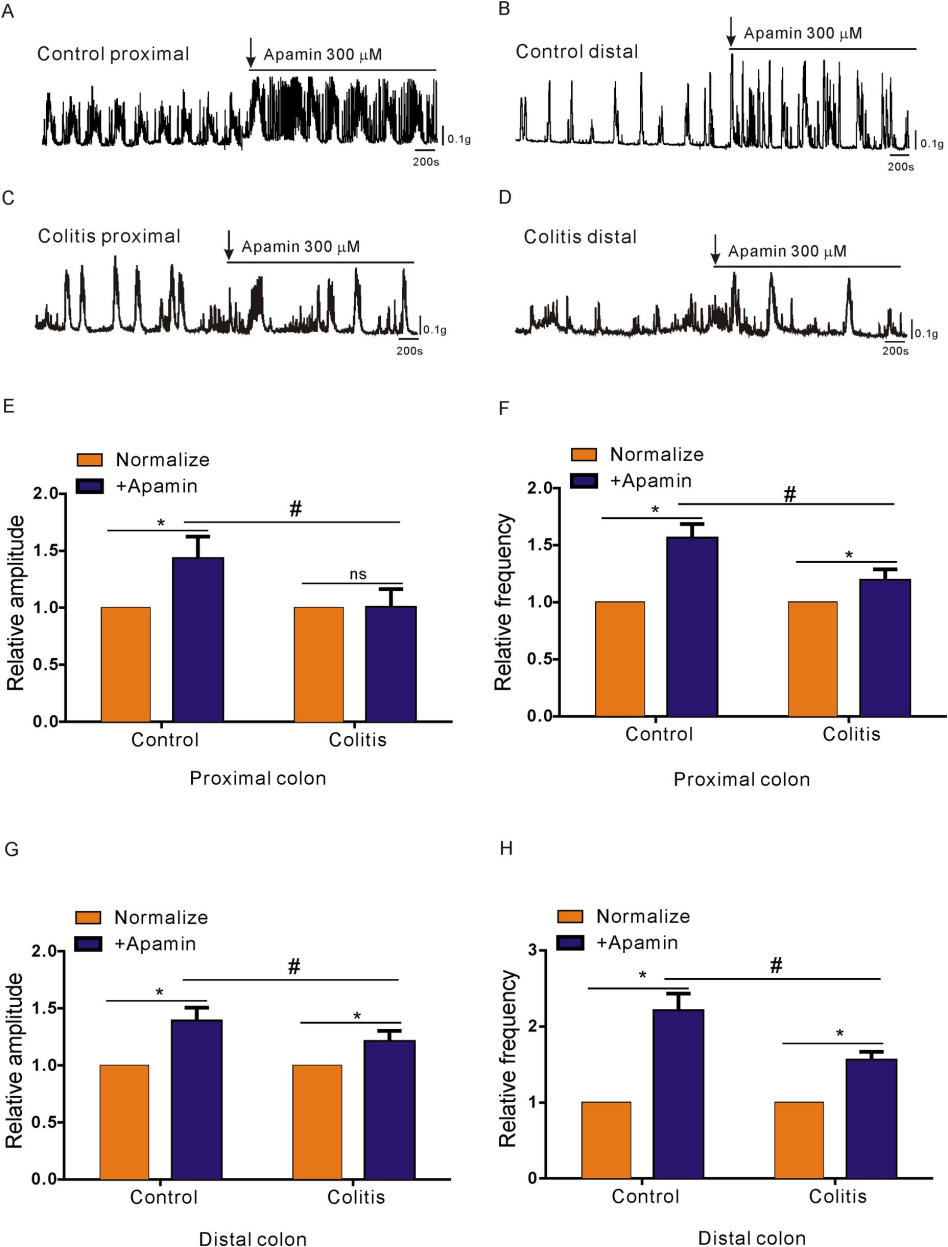
Contractile responses of CMMCs from control and DSS-colitis mice to SK3 antagonists. **A-D.** The excitatory effects of apamin (300 nM) on CMMCs in the proximal and distal colon of both control (A, B) and DSS-colitis mice (C, D). **E-H.** Summary data for the amplitude and frequency of transmission in the proximal (E, F) and distal colons (G, H) of both control and DSS-colitis mice. The data were normalized to the control value (before the application of apamin).

Apamin (300 nM, SK3 channel antagonist) was used in CMMC experiments to further investigate the effects of underexpressed PDGFRα^+^ cells and SK3 channels on colonic transit. The results showed that apamin-treated colitis mice had no obvious drug response, and the contraction intensity of the CMMCs in the proximal colon did not increase significantly, while that of the control group increased markedly (*P < 0.05; n = 7; Fig 3A and 3C). The results indicated that the amplitude and frequency of proximal colon transmission in mice with colitis were significantly lower than those in the control group. (^#^P < 0.05; Fig 3E and 3F). However, in the distal colon, although apamin increased CMMC contraction in colitis mice, apamin significantly enhanced the contraction amplitude and frequency of the distal colon in control mice compared with those in the colitis group (*P < 0.05; n = 7; Fig 3B and 3D). Thus, the contractile intensity and frequency of the distal colon in the mice with colitis were also significantly weaker than those in the control group (^#^P < 0.05; Fig 3G and 3H). These results suggested that SK3 channels played a role in the regulation of colonic transmission. More importantly, SK3 channel function was significantly lower in mice with colitis than in control mice.

### Effect of SK3 channel agonists on CMMCs in PDGFRα^+^ cells

**Fig 4:**
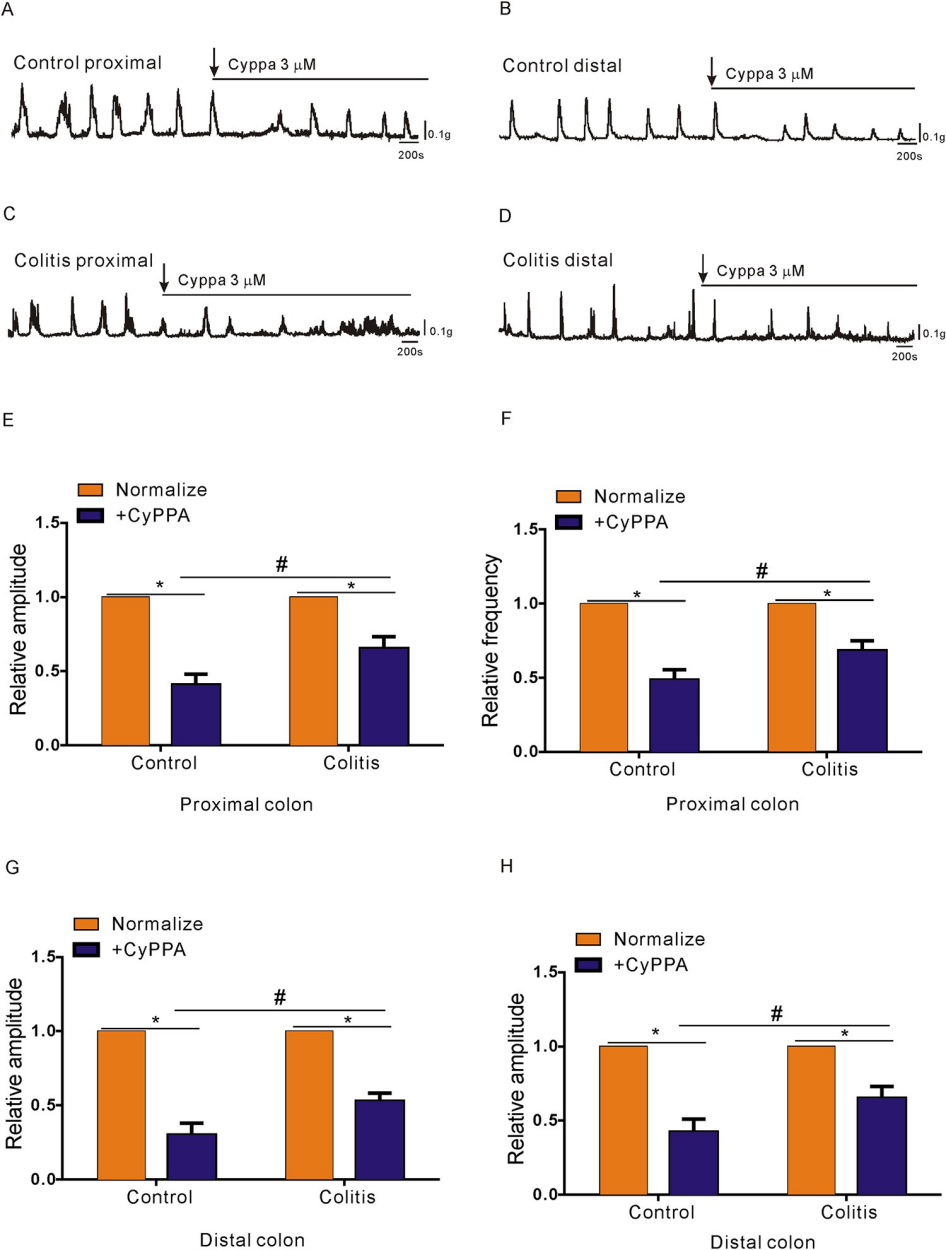
Contractile responses to the SK3 agonist in the CMMCs of control and DSS-colitis mice. **A-D.** Responses of CMMCs to the SK3 agonist (CyPPA, 3 μM) in the proximal and distal colons of both control (A, B) and DSS-colitis mice (C, D). Summary of the CMMC responses to CyPPA, as indicated by the AUC at 400 seconds, in control and DSS-colitis mice. **E-H.** Summary data for the amplitude and frequency in the proximal (E, F) and distal colons (G, H) of both control and DSS-colitis mice. The data were normalized to the control value (before the application of CyPPA).

Subsequently, CyPPA (an SK3 channel agonist; 3 μM) was used in CMMC and smooth muscle contraction experiments. The results revealed that the effect of CyPPA on colonic transit was completely opposite to that of apamin. We observed that CyPPA inhibited CMMC transmission in colitis mice, with proximal contraction decreasing to 58±2.4% and distal contraction decreasing to 55±4.3% (*P<0.05; n = 7; Fig 4C and 4D). In the control group, the inhibitory effect of CyPPA was more significant, reaching 39.6±2.7% in the proximal colon and 25.3±2.4% in the distal colon (*P<0.05; n = 7; Fig 4A and 4B). Notably, the inhibitory effect of CyPPA on colon transmission in the colitis group was weaker than that in the control group (^#^P < 0.05; n = 7; Fig 4E–4H). Thus, we further confirmed that the effect of the SK3 signalling pathway on PDGFRα^+^ cells in colitis mice was decreased; namely, the relaxation effect on smooth muscle was weakened, which may be the main cause of colonic transmission disorders and even diarrhoea in colitis mice.

### Expression of PDGFRα and SK3 in control and colitis mice

**Fig 5:**
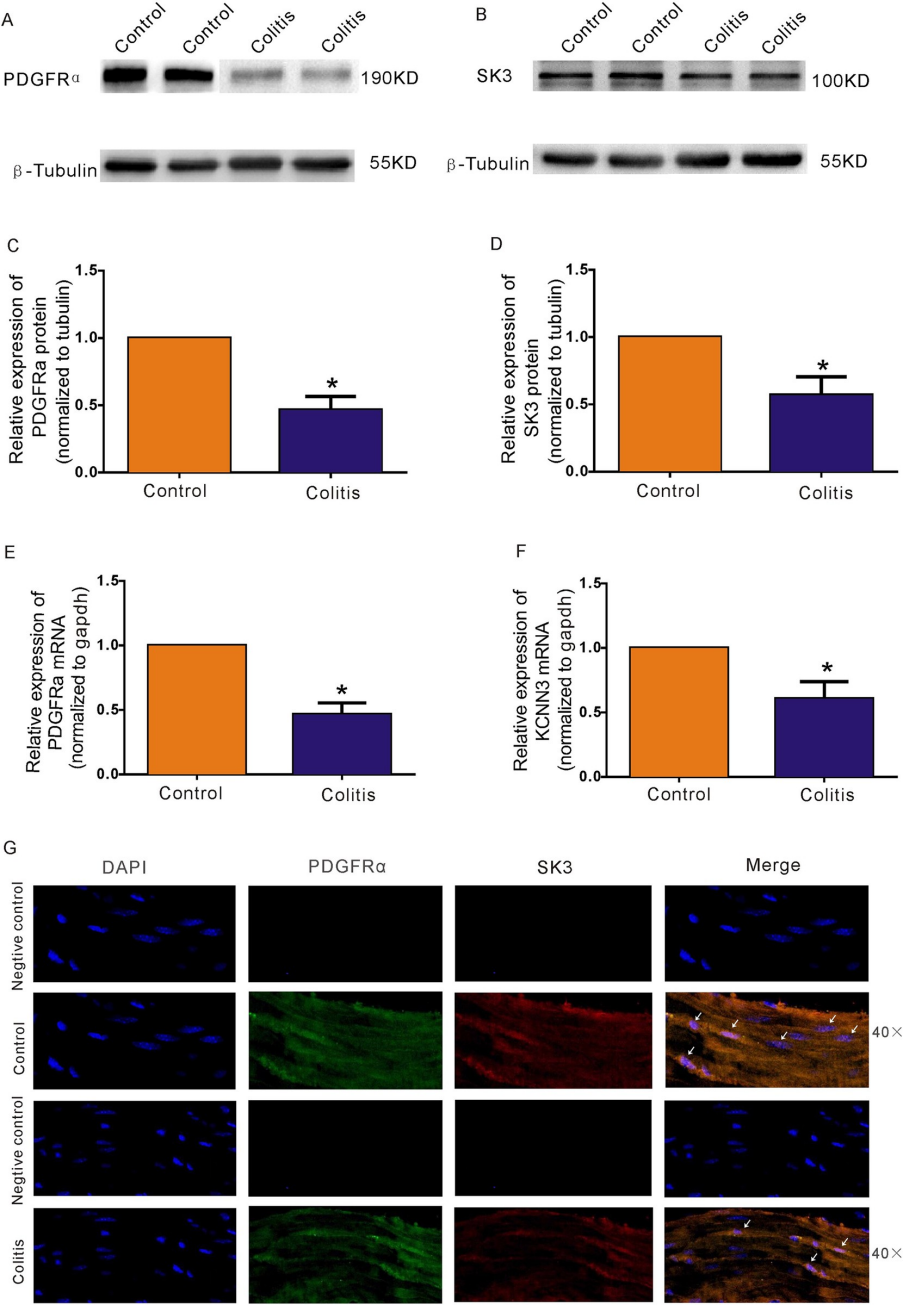
Expression of PDGFRα and SK3 in the colonic muscle layers of control and DSS-induced colitis mice. **A–D.** Western blot analysis of PDGFRα and SK3 in control and DSS-induced colitis mice. The data were analysed using densitometric quantification (% tubulin and normalized to data from control mice; n = 7, *P < 0.05). **E-F.** Quantitative RT‒PCR analysis of PDGFRα and SK3 expression in the colonic muscle layers of control and DSS-induced colitis mice. **G.** SK3 channels colocalized with PDGFRα^+^ cells in the smooth muscle layer in the control and DSS-induced colitis mouse groups. The data were normalized to *gapdh* and the data from control mice (*pdgfr*α n = 7; *kcnn3* n = 8; *P < 0.05).

Based on the assumption that PDGFRα expression reflects the identification of PDGFRα^+^ cells [20], we examined the expression of PDGFRα in the colonic smooth muscle tissue from the colitis experimental group and control group. PDGFRα protein expression was significantly lower in the colonic smooth muscle tissue of colitis mice (46.6 ± 3.7%; P < 0.05; n = 7; Fig 5A and 5C) than in that of control mice. Additionally, according to the transcript level detected using RT‒PCR, the expression of the PDGFRα gene was also 45.7 ± 3.3% lower (P < 0.05; n = 7; Fig 5E) in the colitis mice than in the control mice.

In addition, considering the high expression of SK3 channel proteins in PDGFRα^+^ cells, the protein and gene expression of SK3 were measured using Western blotting and RT‒PCR [6,21]. Compared with that in the control group, the SK3 channel protein level in the colitis group decreased to 56.9±5.1% (P < 0.05; n = 7; Fig 5B and 5D). Moreover, the expression of the *kcnn3* gene, which encodes the SK3 channel, decreased to 60.1±5.0% (P < 0.05; n = 8; Fig 5F). Next, we explored the localization of the SK3 channel to PDGFRα^+^ cells in the colonic smooth muscle layer. The results showed that SK3 channels localized to stellate-shaped or spindle-shaped cells in the smooth muscle tissue, and the number of SK3 channels colocalized PDGFRα^+^ cells in the DSS-colitis mouse group was much lower than that in the control group (Fig 5G). These results indicated that both PDGFRα and SK3 were downregulated in the colon of mice with colitis.

## Discussion

To date, colitis is widely reported to be caused by proinflammatory factors in the mucosa and submucosa, which lead to colonic dysmotility [22–26]. However, few studies have elucidated the myogenic and neurogenic mechanisms of dysdynamic transmission in individuals with colitis from the perspective of the smooth muscle layer. Therefore, this study focused on the role of the SK3 signalling pathway in PDGFRα^+^ cells in colitis-induced colon transit dysmotility. More recently, the growth of intestinal smooth muscle cells was found to be promoted by inflammatory cytokines via the induction of PDGF-Rβ [25]. We know that normal gastrointestinal motility requires the perfect coordination of various cells in the SIP syncytium (including ICCs, PDGFRα^+^ cells, and SMCs). Myogenic contraction is mediated by interstitial cells ICC and PDGFRα^+^ cells, while smooth muscle cells are electrically coupled with these two cells through gap junction, passively contracting and relaxing, and finally pushing feces from the proximal colon to the rectum and anus [8,27]. Furthermore, the purine neurotransmitter released by intestinal motor neurons reduces the excitability of SMC by activating SK3 channel in PDGFRα^+^ cells [18]. Therefore, the change of cell channel current in SIP syncytium may affect the excitability of SMC and eventually lead to gastrointestinal motility disorder. Previous studies have also reported that purinergic and nitrogenous neurotransmitters, rather than acetylcholine, play a key role in neuromuscular activities, thus indicating that inhibitory purinergic signaling pathway plays a leading role in regulating colonic motility transmission [28,29].

In this study, we initially compared the colonic transmission patterns of control and colitis mice by assessing CMMC, the predominant mode of colonic transmission. The results indicated significant differences in both amplitude and frequency of transmission in the colitis group, compared to those in the control group. It is interesting to note that compared with the distal colon of the control group, the contraction intensity of the proximal colon is more obvious, resulting in a pressure gradient from the proximal colon to the distal colon, thus promoting the feces to advance to the rectum and anus (see Fig 2).

Studies have shown that SK3 channel is a key ion channel that is distributed on PDGFRα^+^ cells and mediates the relaxation reaction of PDGFRα^+^ cells. Purine, an inhibitory transmitter released by ENS, can activate SK3 channel through the unique P2Y1 receptor on PDGFRα^+^ cells, causing potassium ion outflow to cause hyperpolarization of PDGFRα^+^ cells, and then hyperpolarization of SMCs through gap junctions, thereby inhibiting the activation of L-type calcium channel on SMCs, and eventually, leading to the relaxation response of colon smooth muscle.

As we found that the distribution and function of PDGFRα^+^ cells and SK3 channels in colonic smooth muscle of colitis mice were significantly down-regulated, agonists and antagonists of SK3 channel in colitis mice were less sensitive to colon transit and smooth muscle contraction than those in the control group. SK3 channels are prominently present in PDGFRα^+^ cells and respond to purines released from enteric neuromuscular bundles, leading to the outward flow of potassium currents. Furthermore, SK3 channels within PDGFRα+cells are crucial for suppressing gastrointestinal motility through purinergic neurotransmission [30,31]. Therefore, antagonists and agonists of SK3 channels were used to study the contributions of dysfunctional colonic transmission in colitis mice. Compared with those in the control group, the enhanced contraction response to apamin during colonic transport and the inhibited transport response to CyPPA in the colitis group were weaker than those in the control group, indicating that the SK3-mediated signalling pathway was significantly downregulated in PDGFRα^+^ cells in the colitis group (Figs 3 and 4). In addition, after apamin was added to the proximal colon to block the SK3 channel, no obvious contraction enhancement was detected, while obvious contraction enhancement was noted in the distal colon (Fig 3C–3F). We believe that this result may be caused by the transmission disorder of the proximal colon in the colitis group; this result may be due to the fact that more PDGFRα^+^ cells are distributed in the distal colon than in the proximal colon. However, further exploration of the expression and functional changes in the SK3 signalling pathway in PDGFRα^+^ cells in the proximal or distal colon is needed. The colitis group exhibited reduced protein and gene expression levels of SK3 and PDGFRα compared to the control group. Furtherm, immunofluorescence results further confirmed the downregulation of the SK3 signaling pathway in PDGFRα^+^ cells in the colitis group compared with the control group, as shown in Fig 5.

These findings revealed that underexpressed SK3 channels decreased diastole function in PDGFRα^+^ cells electrically coupled to SMCs during colonic motility, which induced irregular muscle contraction in the colitis group. Moreover, apamin can block the IJPs elicited by the stimulation of purinergic neurons, inducing obvious depolarization and increasing excitability in colonic SMCs [32–34]. Electrophysiological studies show that ICCs and PDGFRα^+^ cells affect resting potential through gap junction with SMCs, thus regulating the excitability of smooth muscle [8,35]. In this paper, our results show that when the SK3 signalling pathway, which mediates colon diastolic function, is downregulated, the excitability of colon smooth muscle contraction is relatively increased, which may be one of the main factors leading to clinical diarrhoea in patients with colitis, thus providing a new direction for the clinical treatment of patients with diarrhoeal colitis.

PDGFRα^+^ cells are critical targets of purinergic neurotransmitters in gastrointestinal smooth muscle tissue and mediate the fast IJPs induced by inhibitory purinergic signalling. In addition, recent studies have shown that WFDC2, TTLL12, THRA and EPHB3 play a crucial role as key genes of UC-CRC, which are positively correlated with the molecular transformation of UC into CRC. Together, these genes could serve as potential biomarkers and therapeutic targets to combat UC-induced colorectal cancer in humans [36]. Purinergic neurotransmitters coupled with P2Y1 receptor induce hyperpolarization by activating SK3 channel to increase intracellular Ca^2+^ concentration and promote K^+^ outflow, and act on SMCs through gap-linked electrical coupling, which eventually leads to the occurrence of fast IJPs [12,37,38]. Therefore, how the P2Y1 receptor on PDGFRα^+^ cells in individuals with colitis regulates the SK3 signalling pathway through purinergic neurotransmitters will be further explored.

## Conclusions

In summary, colon motility is regulated by both stimulating and suppressing neurons, with the receptors for these neurotransmitters primarily located in the SIP syncytium. In the SIP syncytium, ICC is not only a pacemaker for the autonomic movement of smooth muscle in digestive tract, but also involved in the information transmission between nerves and smooth muscles. There is a very important functional protein ANO1 on ICC cells, which is not only a calcium-activated chloride channel, but also a key ion channel for generating pacemaker current. When ANO1 channel is activated, a large number of chloride ions can flow out, causing depolarization of ICC cells, and then depolarization of SMC through gap junctions, leading to the contraction of smooth muscle. However, PDGFRα^+^ cells induce the relaxation response of smooth muscle through SK3 channels on the cells. The functional down-regulation of SK3 signaling pathway in PDGFRα^+^ cells in colitis group destroyed the coordination between ICC and PDGFRα^+^ cell in SIP syncytium, disrupted the regular colon transit, and finally led to colon transit disorder in colitis mice. Furthermore, this research broadens the existing knowledge on the function of PDGFRα^+^ cells and their specific SK3 channels, offering a potential novel approach for investigating more efficient therapies for colitis.

## Supporting information

S1 File Contains all the supporting figures. (DOCX)
