# Supplementary figures and images for: Colonic dysmotility regulated by downregulation of PDGFRα+ cells / SK3 channel in DSS-induced colitis mice
Source: PLoS One. 2024 Dec 17;19(12):e0312413. doi: 10.1371/journal.pone.0312413 (PMC11651619; doi:10.1371/journal.pone.0312413)

**Figure 5A (Original images)**

**PDGFRα**


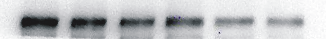


**β-Tubulin**


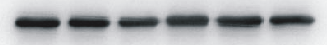


**Figure 5B (Original images)**

**SK3**


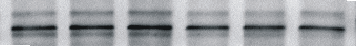


**β-Tubulin**


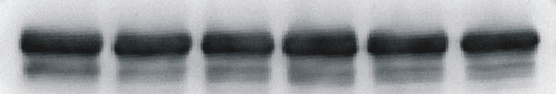


**
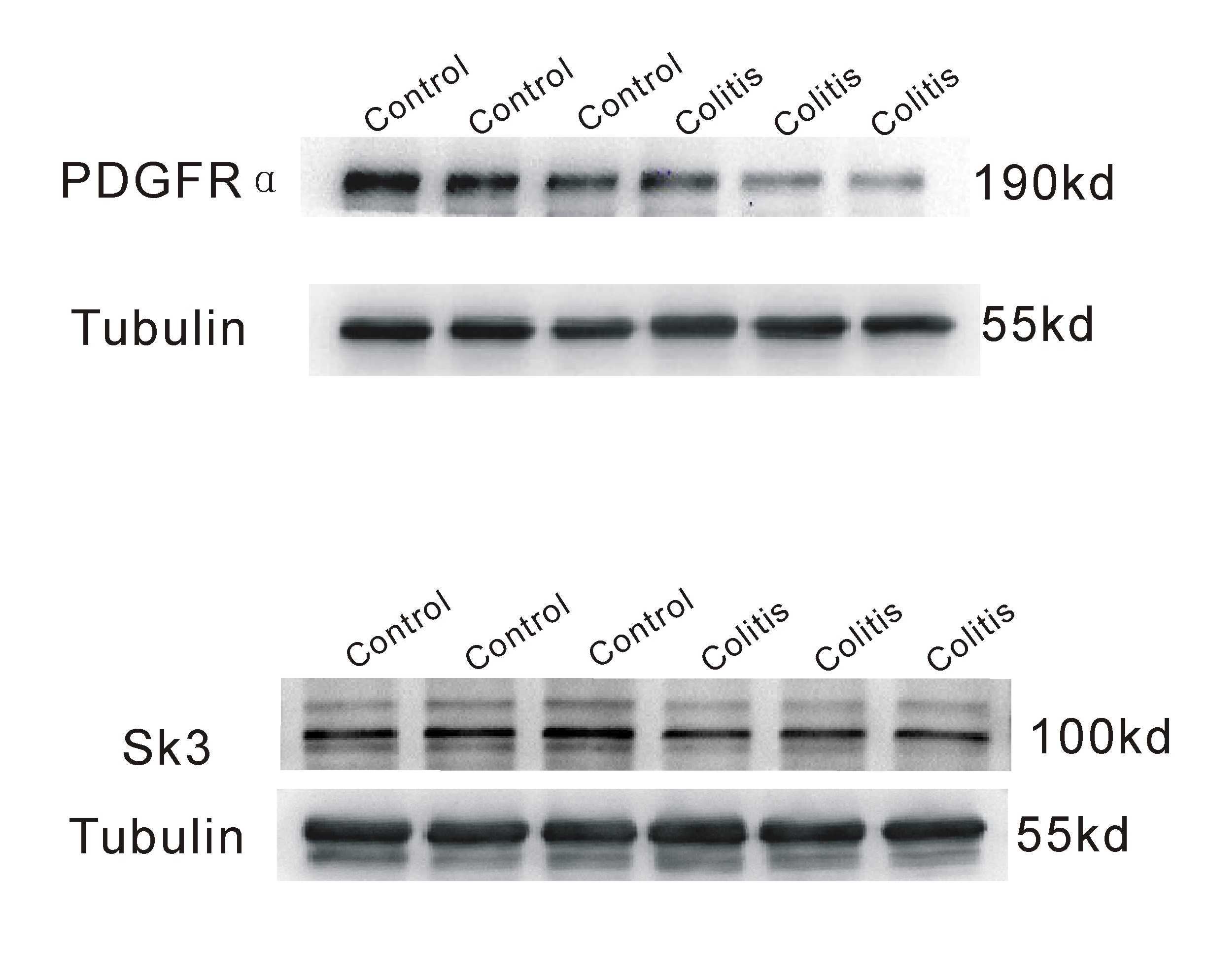
**

Supplement: S1 File — (DOCX) [file pone.0312413.s001.docx]
